# Supplementary material for: Age, sex and primary care setting differences in patients’ perception of community healthcare seeking behaviour towards health services
Source: PLoS One. 2019 Oct 21;14(10):e0224260. doi: 10.1371/journal.pone.0224260 (PMC6802842; doi:10.1371/journal.pone.0224260)
Supplement: Attachment — Submitted filename: Response to reviewers.docx [file pone.0224260.s001.docx]

Manuscript revision v1.0 (PLOS One)

Editor in Chief

PLOS One

Date: 5^th^ September 2019

Dear Dr. Joerg Heber,

**Re: Submission of revised manuscript ([PONE-D-19-17088] - [EMID:51b194bb6104e06b])**

Please find attached a revised version of our manuscript “Age, sex and primary care setting differences in patients’ perception of community healthcare seeking behaviour towards health services”, for publication as a research article in PLOS One.

Your comments and those of the reviewers were highly insightful and enabled us to improve the quality of our manuscript. In the following pages are our point-by-point responses to each of comments/clarification raised by the reviewers.

Revisions in the text are shown using tracked changed. We have enclosed two versions of the manuscript: one with tracked changes and another which is the final unmarked copy. We hope that the revisions in the manuscript and our accompanying responses will be sufficient to make our manuscript suitable for publication in PLOS One.

We shall look forward to hearing from you.

Yours sincerely,

Lim Ming Tsuey

Corresponding author

Centre for Clinical Outcomes Research, Institute for Clinical Research

National Institutes of Health (NIH), Ministry of Health Malaysia

No.1, Jalan Setia Murni U13/52

Seksyen U13, Bandar Setia Alam

40170 Shah Alam

Selangor, Malaysia

Email: mtlim2000@yahoo.com

**Responses to comments from Reviewer #1**

| **No** | **Reviewer comment** | **Response** |
| --- | --- | --- |

| 1 | Have the authors made all data underlying the findings in their manuscript fully available?  No | Yes, we have consulted the principal investigator of the data source and agreed to include our de-identified dataset with relevant variables. As such our data availability statement will be amended to  All relevant data are within the manuscript and its Supporting Information files. |
| --- | --- | --- |
| 2 | Review Comments to the Author  The scientific value of this manuscript is questionable and I don't think that it will have positive impact in the field | We appreciate the reviewer’s concern.  We would like to reassure the reviewer that our findings in this paper would help in   - the understanding of healthcare utilisation pattern by type of health concerns - developing of healthcare policy and planning for effective treatment and appropriate interventions for management of health concerns.  Furthermore, early recognition presentation to health care facilities and compliance with effective treatment have shown to reduce morbidity and thereby mortality [1-3].  1. Van der Hoeven M, Kruger A, Greeff M. Differences in health care seeking behaviour between rural and urban communities in South Africa. International Journal for Equity in Health 2012; 11: 31. 2. Hausmann-Mueala S, Muela Ribera J, Nyamongo I: Health-seeking behaviour and the health system response. Disease Control Priorities Project (DCPP). Working Paper no.14;. 2003 3. World Health Organization: Rapid assessment of health seeking behaviour in relation to sexual transmitted disease: draft protocol.1995 |

**Responses to comments from Reviewer #2**

| **No** | **Reviewer comment** | **Response** |
| --- | --- | --- |

| **1** | Review Comments to the Author  The publication is a good one with much variability's taken and can be accepted from my point of view, but there are some points which must be taken into consideration:  1. Try to avoid repetition of the same sentences as in the abstract (Healthcare seeking ...) within the same paragraph.  2. In some paragraphs you left spaces at the beginning in others not. Please unify  3. In the tables, they are very long. It is advisable to separate them each for a criteria, so that you would have precise characters within one table to avoid long tables for the reader. | We thank the reviewer for his comments and suggestions.  We rephrased the following paragraph to avoid repetitions of same sentence as in the introduction section which read previously  “Healthcare seeking behaviour is also influenced by various determinants such as sex, age, social status, type of illness, access to services and perceived quality of the service [1, 2]. Understanding these potential determinants of healthcare seeking behaviour will be necessary in improving healthcare utilisation and health outcomes within different populations.”  to  Introduction , page 4, line 78-82  Studies have shown that various determinants such as sex, age, social status, type of illness, access to services and perceived quality of the service [1, 2] affect an individual’s healthcare seeking behaviour but findings have been inconsistent. As such, it is necessary to understand these potential determinants in improving healthcare utilisation and health outcomes within different populations.  We have removed the space at the beginning of every paragraph. To differentiate between paragraphs, we inserted an empty line between paragraphs  We acknowledge the concerns raised by the reviewer. However, we believe that the characteristics listed within Table 1 would all complement each other to provide baseline characteristics of our study participants.  We agree that the table is rather long and have amended it. We rephrased the question into more concise phrase.  Kindly refer to Table 1 within the manuscript.  Results, page 11-13, line 247-249 |
| --- | --- | --- |

**Response to comments from Reviewer #3**

| **No** | **Reviewer comment** | **Response** | |
| --- | --- | --- | --- |
| 1 | Have the authors made all data underlying the findings in their manuscript fully available?  No | | Yes, we have consulted the principal investigator of the data source and agreed to include our de-identified dataset with relevant variables. As such our data availability statement will be amended to  All relevant data are within the manuscript and its Supporting Information files. |
| 2 | Review Comments to the Author  Congratulations to the authors for this interesting manuscript regarding the perceived community healthcare seeking behavior for acute and preventive physical and psychosocial health concerns in Malaysian primary care clinics. The authors took into consideration sex, age and affordability of care (health insurance) as key elements of healthcare seeking behaviors in Malaysian community. This is a continuation of the efforts in the previous publications, Quality and Costs of Primary Care(QUALICOPC) study in Malaysia: Phase I –Public Clinics, and Quality and Costs of Primary Care (QUALICOPC) Malaysia: Phase II – Private Clinics (cited as references 12, 13) in understanding potential determinants of community healthcare seeking behaviors in Malaysia.  Comments: 1) Line 116: The data for this study was extracted from the International Quality and Cost of Primary Care (QUALICOPC) study: Can you please add a citation for this statement? you may cite the data used in the study if previously published, alongside with references 12 and 13.  2) Lines 124-125: Further details on the methods of this study are described elsewhere: can you please explain more what these details are for the reader? Did you mean sampling frame, sampling methods, and eligibility of participants?    3) Lines 128-134: Ten patients aged 18 years old and above from each clinic were invited to participate in the survey: The sample size is 3979 patients. Total number of clinics is 221+239= 460 clinics. Overall response rate for both patient questionnaires was 91.1%. One patient will be administered the patient values questionnaire while nine will be administered the patient experience questionnaire. Since the overall sample size was 3979, can you please clarify if there were any excluded patients for incomplete data or because of the exclusion criteria?  4) Lines 129-130: the patient values questionnaire and patient experience questionnaire were mentioned before the appearance of reference 15 in Survey Tool part (which can be considered as a reference for them too).  5) 337-339: please add references to this statement (you can use one of those mentioned later on in this paragraph).  6) Lines 385- 388: A comparison between urban and rural clinics visitors in seeking medical attention for psychosocial or mental health complaints, in addition to acute and preventive physical health concerns, can be considered.  7) Some minor writing errors within the text are present. It is suggested to carefully review the manuscript towards spelling and/or grammatical errors. Some examples are given below:  Lines 55-56: lower than for physical health concerns.  Line 92: [8] while > [8], while  Line 145: were presented > was presented (since the verb is for the sub-section, not the 12 items)  Line 225: they do not their have own general practitioners > do not have their own  Line 406: behaviour [10. > behaviour [10]. | | We thank the reviewer for his comments and suggestions.  We have added the references as follows:  Materials and methods, page 5, line 118-119.  The data for this study was extracted from the International Quality and Cost of Primary Care (QUALICOPC) study conducted in the primary care setting in Malaysia [12, 13].  Yes, and we have amended the sentence. It now reads:    Materials and methods, page 6, line 127-129  Further details on the methods including study design, sampling methods, questionnaires and eligibility criteria are described elsewhere [12, 13].  Thank you  We added the following statements and hope that it would provide better clarity on the number of respondents.  Materials and methods, page 6, line 137-141  A total of 4983 patients were invited and the overall response rate for both patient questionnaires was 91.1%. A non-response analysis was not necessary as it is usually conducted only when response rates fall below 80% [15]. One hundred and three respondents were excluded because of incomplete data. Of the two types of questionnaires administered, we used data from only the patient experience survey in this analysis.  We inserted the required reference as suggested as follows:  Materials and methods, page 6, line 133-134  One patient will be administered the patient values questionnaire while nine will be administered the patient experience questionnaire [14].  We inserted the required references as suggested as follows:    Discussion, page 22, line 350-352  This finding upholds those from previous studies, where women were known to consult their general practitioners more often than men and were more proactive in health seeking [22-23].  22) Thompson AE, Anisimowicz Y, Miedema B, Hogg W, Wodchis WP, Bassler KA. The influence of gender and other patient characteristics on health care-seeking behaviour: a QUALICOPC study. *BMC Family Practice* 2016; 17: 38.  23) Mackenzie CS, Gekoski WL, Knox VJ. Age, gender, and the underutilization of mental health services: the influence of help-seeking attitudes. *Aging Ment Health* 2006; 10: 574–582.  We included a comparative study to support our finding on help-seeking behaviour for mental health problem between rural and urban population.  Discussion, page 24, line 401-406  However, the prevalence of perceived community health seeking for psychosocial concerns is lower compared to results from another Malaysian study which surveyed patterns of help-seeking for common mental disorders within an urban population [36]. Similar finding was also observed in a comparative study where rural residents with mental health problems were less likely to seek help than their urban counterparts [37].  36) Ismail SIF. Patterns and risk factors with help- seeking for common mental disorders in an urban Malaysian community. London School of Hygiene and Tropical Medicine, 2011  37) Caldwell TM, Jorm AF, Dear KBG. Suicide and mental health in rural, remote and metropolitan areas in Australia. The Medical Journal of Australia 2004; 181: S10.  Thank you for highlighting the spelling and/or grammatical errors. We have corrected those mistakes as follow:  Abstract, page 3, line 53-56  Our findings showed that sex and healthcare affordability differences were present in perceived community healthcare seeking behaviour towards primary care services. Also, perceived healthcare seeking behaviour was consistently lower for psychosocial health concerns compared to physical health concerns.    Introduction, page 4, line 92-94  Thus far, patient surveys on healthcare seeking behaviour have often focused on disease specific areas such as tuberculosis [7] or depression [8], while less attention has been given to primary care in general.  Introduction, page 7, line 152-154  Only the sub-section on the community healthcare seeking behaviour perception (12 items) from the patient experience questionnaire was presented in this analysis and all patients who responded to this questionnaire were included.  Results, page 10, line 239-240  About 80% of patients claimed that they do not have their own general practitioners to first consult on a health problem.  Discussion, page 25, line 424-426  While majority of studies on primary healthcare seeking behaviour have investigated utilisation patterns or individual health behaviours, few have looked at the perceived community healthcare seeking behaviour [10]. |

Additional information (From Ms Michelle Ellis)

| **No** | | **Enquiry** | **Response** | |
| --- | --- | --- | --- | --- |
| 1 | Please include a copy of the interview guide used in the study, in both the original language and English, as Supporting Information, or include a citation if it has been published previously | | | The copy of patient questionnaires in English, Malay and Mandarin languages are available at the citation as mentioned in following section of the manuscript  Materials and methods, page 6, line 127-129  Further details on the methods including study design, sampling methods, **questionnaires** and eligibility criteria are described elsewhere [12, 13]. |
