# Supplementary material for: Age, sex and primary care setting differences in patients’ perception of community healthcare seeking behaviour towards health services
Source: PLoS One. 2019 Oct 21;14(10):e0224260. doi: 10.1371/journal.pone.0224260 (PMC6802842; doi:10.1371/journal.pone.0224260)
Supplement: Attachment — Submitted filename: Response to reviewers.docx [file pone.0224260.s002.docx]

Manuscript revision v1.2 (PLOS One)

Editor in Chief

PLOS One

Date: 26^th^ September 2019

Dear Dr.Joerg Heber,

**Re: Submission of revised manuscript ([PONE-D-19-17088R1] - [EMID:** **abf2406225040f7c])**

Please find attached a revised version of our manuscript “Age, sex and primary care setting differences in patients’ perception of community healthcare seeking behaviour towards health services”, for publication as a research article in PLOS One.

Your comments and those of the reviewers were highly insightful and enabled us to improve the quality of our manuscript. In the following pages are our point-by-point responses to each of comments/clarification raised by the reviewers.

Revisions in the text are shown using tracked changed. We have enclosed two versions of the manuscript: one with tracked changes and another which is the final unmarked copy. We hope that the revisions in the manuscript and our accompanying responses will be sufficient to make our manuscript suitable for publication in PLOS One.

We shall look forward to hearing from you.

Yours sincerely,

Lim Ming Tsuey

Corresponding author

Centre for Clinical Outcomes Research, Institute for Clinical Research

National Institutes of Health (NIH), Ministry of Health Malaysia

No.1, Jalan Setia Murni U13/52

Seksyen U13, Bandar Setia Alam

40170 Shah Alam

Selangor, Malaysia

Email: mtlim2000@yahoo.com

**Responses to comments from Reviewer #1**

| **No** | **Reviewer comment** | **Response** |
| --- | --- | --- |

| 1 | Review Comments to the Author  I want to thank the authors for their effort in this manuscript entitled "Age, sex and primary care setting differences in patients’ perception of community healthcare seeking behaviour towards health services'. With all my respect to the authors, again the scientific value of this manuscript is questionable and I don't think that it will have a positive impact in the field. My comments: First: I think the sub-section (12 items) of the main tool of QUALICOPC only can’t measure the community health seeking behavior. Second: The main question is “Would most people visit a clinic doctor for?”. In my opinion, the question is asking the patients what other people expected to do. I can talk for myself, not about what others think. | We appreciate the reviewer’s comments. We agree that evaluating patient’s health seeking would necessitate a direct evaluation of the patient. We also agree with the reviewer that we were not directly measuring the community healthcare seeking behaviour. Nevertheless, we are taking a different angle. We apologise for not being sufficiently clear enough. We were evaluating our primary health care responsiveness to community needs. Thus the focus is on *community perception* rather than patient direct health seeking behaviour.  The objective of the survey question for the perceived health seeking behaviour is regarding the perceptions of patients about conditions warranting a visit to our primary care services. As such, the main question is designed as *“Would most people visit a clinic doctor for?”.* This refers to what a person perceives the community would seek in terms of healthcare.  Therefore, we have revised some of sentences in the introduction section of our manuscript and hope that it would provide better clarity on our objective which is to describe community perception of healthcare seeking behaviour.  Introduction, page 4, line 88-90,  In this study, we focus on **perceived community** healthcare seeking behaviour in primary care as primary care is often patients’ first point of contact with the healthcare system for most medical problems.  Introduction, page 4-5, Line 94-101:  **Understanding this is important because the community perception provides another indicator of primary care utilisation**. We aim to bridge this gap in knowledge on healthcare seeking behaviour which will not only reflect the patterns of **perceived** healthcare seeking behaviour at the different stages of utilisation, access and barriers to healthcare services in a given community but also the potential **determinants to community perceptions**. [9]. Hence, **perceived** healthcare seeking behaviour can serve as a tool to understand how healthcare services are used and also to gauge future demand for healthcare services.  We believe this patients’ perception of community of healthcare seeking behaviour study is important as it reflects the ‘Comprehensiveness of services’ where patients’ views on the breadth of the clinical task proﬁle of services oﬀered by the GP are taken into consideration.  Our findings of this study would hopefully have direct implications to practice and healthcare policy maker such as efforts to increase awareness of available healthcare services especially in psychosocial health concerns should be made to address this gap in healthcare, to improve barriers to health care access by identifying those who are more likely to engage in health care-seeking behaviours and the variables predicting health care-seeking and consequently, those who are not accessing primary care can be targeted and policies can be developed and put in place to promote their health care-seeking behaviour.  In addition, previous studies have demonstrated that information gathered on health care seeking behaviour would not only help in the understanding of healthcare utilisation pattern by type of health concerns but also crucial for developing health care policies and planning for early diagnosis, effective treatment and appropriate interventions [1-3].  1. Van der Hoeven M, Kruger A, Greeff M. Differences in health care seeking behaviour between rural and urban communities in South Africa. International Journal for Equity in Health 2012; 11: 31.  2. Hausmann-Mueala S, Muela Ribera J, Nyamongo I: Health-seeking behaviour and the health system response. Disease Control Priorities Project (DCPP). Working Paper no.14; 2003.  3. World Health Organization: Rapid assessment of health seeking behaviour in relation to sexual transmitted disease: draft protocol 1995. |
| --- | --- | --- |
